# Cannabinoids in Glioblastoma Therapy: New Applications for Old Drugs

**DOI:** 10.3389/fnmol.2018.00159

**Published:** 2018-05-16

**Authors:** Claudia A. Dumitru, I. Erol Sandalcioglu, Meliha Karsak

**Affiliations:** ^1^Department of Neurosurgery, KRH Klinikum Nordstadt, Nordstadt Hospital Hannover, Hannover, Germany; ^2^Neuronal and Cellular Signal Transduction, Center for Molecular Neurobiology Hamburg (ZMNH), University Medical Center Hamburg-Eppendorf (UKE), Hamburg, Germany

**Keywords:** cannabinoids, glioblastoma, molecular mechanisms, novel therapeutic strategies, cannabinoid receptors, cannabidiol, THC

## Abstract

Glioblastoma (GBM) is the most malignant brain tumor and one of the deadliest types of solid cancer overall. Despite aggressive therapeutic approaches consisting of maximum safe surgical resection and radio-chemotherapy, more than 95% of GBM patients die within 5 years after diagnosis. Thus, there is still an urgent need to develop novel therapeutic strategies against this disease. Accumulating evidence indicates that cannabinoids have potent anti-tumor functions and might be used successfully in the treatment of GBM. This review article summarizes the latest findings on the molecular effects of cannabinoids on GBM, both *in vitro* and in (pre-) clinical studies in animal models and patients. The therapeutic effect of cannabinoids is based on reduction of tumor growth via inhibition of tumor proliferation and angiogenesis but also via induction of tumor cell death. Additionally, cannabinoids were shown to inhibit the invasiveness and the stem cell-like properties of GBM tumors. Recent phase II clinical trials indicated positive results regarding the survival of GBM patients upon cannabinoid treatment. Taken together these findings underline the importance of elucidating the full pharmacological effectiveness and the molecular mechanisms of the cannabinoid system in GBM pathophysiology.

## Glioblastoma

Gliomas are the most common primary tumors of the central nervous system. Half of the newly-diagnosed gliomas are glioblastomas (GBMs), with an incidence in adults of 0.59–3.69 cases per 100,000 person life-years (Ostrom et al., 2014). The vast majority of GBM develop *de novo* (primary GBM); however, GBM can also evolve from lower grade gliomas (secondary GBM). Primary GBM occur more commonly in male patients whereas the reverse is the case for secondary GBM (Adamson et al., 2009). The mean age of primary and secondary GBM patients is 62 and 45 years, respectively (Adamson et al., 2009).

GBM is an extremely aggressive type of cancer. These tumors are characterized by high cellular proliferation and angiogenesis resulting in rapid tumor growth and, consequently, necrosis. GBM cells also exhibit high migration and invasive properties, which allow them to produce metachronous lesions and even to spread through the brain parenchyma. Furthermore, GBM tumors contain a subpopulation of glioma stem-like cells (GSCs), which, at least partially, account for the high resistance to therapy and recurrence rates of these tumors (Louis et al., 2016).

Currently, the standard of care treatment for GBM consists of maximum safe surgical resection followed by radiotherapy plus concomitant and adjuvant chemotherapy with temozolomide (TMZ; Stupp et al., 2005). Despite this aggressive therapeutic regimen, GBM patients have a poor prognosis, with only 0.05%–4.7% of patients surviving 5 years past initial diagnosis (Ostrom et al., 2014). Recent advances in molecular pathology identified various GBM subtypes and thus, paved the way for more individualized therapeutic strategies. However, GBM remains incurable at present and there is still an urgent need to further characterize and target the molecular mechanisms involved in its progression.

## Cannabinoids

The term “cannabinoids” originally described bioactive constituents of the plant *Cannabis sativa*. The cannabis ingredients were used traditionally for their medicinal purpose but also for their recreational properties. In addition to the psychoactive cannabinoid Δ^9^-tetrahydrocannabinol (THC), a number of other phytocannabinoids have been successfully extracted such as cannabinol, cannabidiol (CBD), cannabigerol or the flavoring agent beta-caryophyllene (BCP; Mechoulam, 1970; Gertsch et al., 2008). Most of the cannabinoids bind to G-protein coupled cannabinoid receptors, CB1 and CB2, and act as agonists or inverse agonists. Of special interest for therapeutic purposes are cannabinoids that are absent of intoxicating effects such as the CB2-selective BCP and CBD (Sharma et al., 2016; Russo, 2017). The cannabis constituent CBD has no significant agonistic activity on cannabinoid receptors (Howlett et al., 2002; Pertwee, 2005) however it targets a number of G-protein coupled receptors like GPR12, GPR6, GPR3, GPR55 and 5-HT1A and also transient receptor potential vanilloid TRPV1 and TRPV2 (Espejo-Porras et al., 2013; Nabissi et al., 2013; Hassan et al., 2014; Brown et al., 2017; Kaplan et al., 2017; Laun and Song, 2017). Cannabinoid receptors can also be selectively activated by pharmacologically efficient synthetic cannabinoids. Furthermore, cannabinoid receptors are activated by endogenously-produced arachidonic acid derivatives. The so-called endocannabinoids, anandamide and 2-arachidonoylglycerol (2-AG), are synthesized from cell membrane phospholipids by specific enzymes. In GBM, increased levels of anandamide and reduced activity of the synthesizing enzyme N-acylglycerol phosphatidylethanolamine–phospholipase D (NAPE-PLD) and degrading enzyme fatty acid amide hydrolase (FAAH) have been identified (Petersen et al., 2005).

The activation of G-alpha i/o-coupled cannabinoid receptors inhibits adenylate cyclases, signals via ceramide, and induces kinase phosphorylation of focal adhesion kinase (FAK), mitogen-activated protein kinase (MAPK), and phosphatidylinositol-3-kinase (PI3K). Cannabinoid receptors also regulate the expression of immediate early genes and regulate the production of nitric oxide (Howlett et al., 2002). Additionally, certain voltage dependent calcium and inwardly rectifying potassium channels can be modulated via cannabinoid receptor signaling (Lu and Mackie, 2016). Thus, activation of CB1 or CB2 receptors exerts diverse consequences on cellular biology and functions (Lu and Mackie, 2016).

## Molecular Mechanisms of Cannabinoids in GBM

GBM tumors are known to express both major cannabinoid-specific receptors CB1 and CB2. The expression of these receptors has been detected in GBM cell lines, in *ex-vivo* primary tumor cells derived from GBM patients and *in situ*, in GBM tissue biopsies. There is a general consensus that high-grade gliomas, including GBM, express high levels of CB2. Furthermore, CB2 expression positively correlates with the malignancy grade (reviewed in Ellert-Miklaszewska et al., 2013). In contrast, the expression of CB1 still requires characterization, as it has been reported to be either unchanged (Schley et al., 2009), decreased (De Jesús et al., 2010) or even increased (Wu et al., 2012; Ciaglia et al., 2015) in GBM compared to low-grade gliomas or non-tumor control tissues.

**Figure 1:**
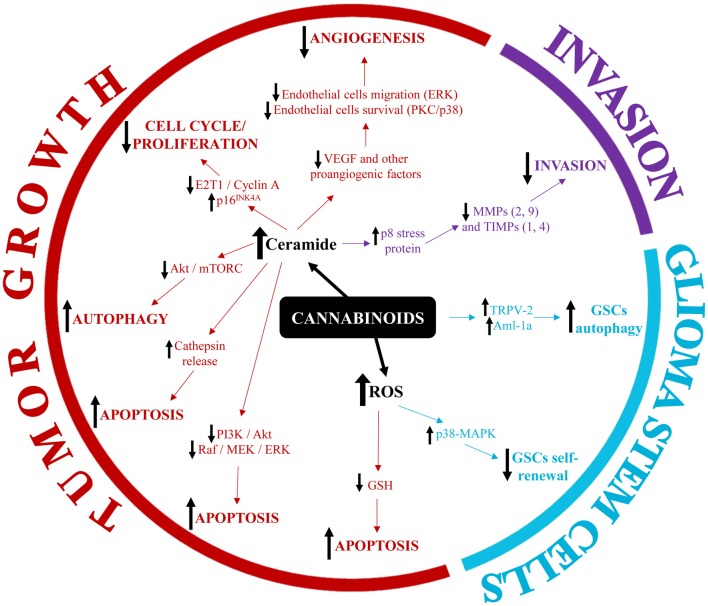
Summary of the main molecular mechanisms involved in the modulation of glioblastoma (GBM) pathophysiology by cannabinoids.

The identification of altered expression of cannabinoid receptors in gliomas and GBM led to the hypothesis that cannabinoid receptor agonists might be used as anticancer agents. Indeed, a pilot clinical study was already developed more than a decade ago to investigate the anti-tumor activity of THC in patients with glioma. The study held promising results as it showed a decrease of tumor cell proliferation upon administration of THC in two of nine patients (Guzmán et al., 2006). Since then, an increasing number of studies sought to elucidate the molecular mechanisms triggered via the cannabinoids-cannabinoid receptors axis in gliomas and GBM. The major findings are described below and a summary is provided in Figure 1.

## Cannabinoids and GBM Tumor Growth

The best studied effect of cannabinoids on GBM pathophysiology is the inhibition of tumor growth. A number of *in vivo* studies demonstrated that cannabinoids could significantly reduce tumor volume in orthotopic and subcutaneous animal models of glioma (for a comprehensive review, see Rocha et al., 2014). The mechanisms mediating this phenomenon can be roughly grouped into three categories: (1) cell death-inducing mechanisms (apoptosis and cytotoxic autophagy); (2) cell proliferation-inhibiting mechanisms; and (3) anti-angiogenic mechanisms.

Cannabinoid-induced cell death occurs mainly through the intrinsic (mitochondria-dependent) apoptotic pathway (reviewed in Ellert-Miklaszewska et al., 2013). Briefly, the pro-apoptotic Bcl-2 family member Bad is phosphorylated in response to cannabinoid treatment and translocates to the mitochondria. This results in loss of integrity of the outer mitochondrial membrane, release of cytochrome c and activation of apoptosis-executioner caspases. The activation of the intrinsic apoptosis pathway by cannabinoids is thought to be mediated by an increase in intracellular ceramide which, in turn, inhibits the pro-survival pathways PI3K/Akt and Raf1/MEK/ERK thereby allowing Bad to translocate to the mitochondria. Interestingly, ceramide has been also implicated in cannabinoid-induced autophagy of glioma cells through the p8/TRB3 pathway and subsequent inhibition of the Akt/mTORC1 axis (Carracedo et al., 2006; Salazar et al., 2009). Recent studies additionally showed that THC altered the balance between ceramides and dihydroceramides in autophagosomes and autolysosomes, which promoted the permeabilization of the organellar membrane, the release of cathepsins in the cytoplasm and the subsequent activation of apoptotic cell death (Hernández-Tiedra et al., 2016).

In addition to ceramide-mediated cell death, cannabinoids were also shown to trigger apoptosis via oxidative stress (reviewed in Massi et al., 2010). Specifically, glioma cells treated with CBD responded with reactive oxygen species (ROS) production, GSH depletion and caspase-9, -8 and -3 activation. Furthermore, combined treatment of GBM cells with THC and CBD induced a significant increase in the formation of ROS, which was linked to a later induction of apoptosis (Marcu et al., 2010). Recently however, Scott et al. (2015) showed that, while CBD treatment of glioma cells did induce a significant increase in ROS production, this phenomenon was accompanied by an upregulation of a large number of genes belonging to the heat-shock protein (HSP) super-family. As the subsequent upregulation of HSP client proteins diminished the cytotoxic effect of CBD, the authors proposed that the inclusion of HSP inhibitors might enhance the anti-tumor effects of cannabinoids in glioma/GBM treatment regimens (Scott et al., 2015).

Apart from a direct killing effect on tumor cells, cannabinoids can also induce cell cycle arrest thereby inhibiting tumor cell proliferation. For instance, treatment of GBM cells with THC and/or CBD increases the population of cells in the G_0_-G_1_ phase and G_2_-G_M_ phase while decreasing the number of cells in the S-phase (Marcu et al., 2010). Similarly, Galanti et al. (2008) found that administration of THC to human GBM cell lines induced G_0_-G_1_ phase arrest. The authors also characterized some of the molecular mechanisms involved in cannabinoid-induced cell cycle arrest and found that THC decreased the levels of E2F1 and Cyclin A (two proteins that promote cell cycle progression) while upregulating the level of the cell cycle inhibitor p16^INK4A^ (Galanti et al., 2008).

The inhibitory effects of cannabinoids on GBM growth are, however, not restricted to the direct modulation of tumor cell death/survival or proliferation pathways. Several studies showed that cannabinoids were also able to inhibit tumor angiogenesis. For instance, Blázquez et al. (2003) found that local administration of the nonpsychotic cannabinoid JWH-133 to mice inhibited angiogenesis of malignant gliomas, since the cannabinoid-treated tumors had a small, differentiated and impermeable vasculature while the vasculature of the control tumors was large, plastic and leaky (Blázquez et al., 2003). The same group later demonstrated that local administration of THC resulted in a decrease of pro-angiogenic VEGF levels in two patients with recurrent GBM (Blázquez et al., 2004). *In vitro*, cannabinoids inhibited endothelial cell migration via the ERK pathway and endothelial cell survival via protein kinase C (PKC) and p38-MAPK pathways (Blázquez et al., 2003). Similarly, Solinas et al. (2012) demonstrated that CBD induced endothelial cell cytostasis, inhibited endothelial cell migration and sprouting *in vitro* and inhibited angiogenesis *in vivo*. These effects were accompanied by a downregulation of pro-angiogenic factors such as matrix metalloprotease-2 and -9 (MMP2 and MMP9), urokinase-type plasminogen activator (uPA), endothelin-1 (ET-1), platelet-derived growth factor-AA (PDGF-AA) and chemokine (c-x-c motif) ligand 16 (CXCL16; Solinas et al., 2012).

While most studies found that the agonistic stimulation via CB receptors is responsible for the anti-tumor effects of cannabinoids, recent evidence suggests that CB1 antagonists might also be useful in glioma therapy. Specifically, Ciaglia et al. (2015) found that the pharmacological inactivation of CB1 by SR141716 inhibited glioma cell growth through cell cycle arrest and induction of caspase-dependent apoptosis. Interestingly however, SR141716 additionally upregulated the expression of NKG2D ligands (MICA and MICB) on the surface of glioma cells via STAT3 inactivation. The increase of MICA/B levels subsequently enhanced the recognition and killing of glioma cells by NK-cells. Notably, SR141716-induced MICA/B upregulation directly correlated with the degree of CB1 expression and occurred only in malignant glioma cells but not in normal human astrocytes (Ciaglia et al., 2015). Taken together these findings suggest that CB1 specific antagonists might be useful in multimodal therapeutic strategies, at least for certain subsets of GBM with high expression of CB1.

## Cannabinoids and GBM Invasion

Although gliomas and GBM rarely metastasize, these tumor cells are very adept at infiltrating the surrounding healthy brain tissue and spreading through the brain parenchyma (reviewed in Manini et al., 2018). Therefore, therapeutic strategies aimed at inhibiting the migration and invasion of GBM cells are of great clinical relevance in the management of this disease.

The role of cannabinoids in GBM migration and invasion is still poorly characterized. Nevertheless, accumulating evidence suggests that cannabinoids have potent anti-invasive effects on glioma cells both *in vitro* and *in vivo*. For instance, Soroceanu et al. (2013) showed that CBD inhibited the invasion of GBM cells through organotypic brain slices. This anti-invasive effect was attributed to the inhibition of Id-1 expression by CBD and was observed in several GBM cell lines, in *ex-vivo* primary GBM cells and in an orthotopic xenograft murine model (Soroceanu et al., 2013). Solinas et al. found that CBD significantly inhibited GBM invasion even at low concentrations, which were otherwise not sufficient to induce tumor cell death (Solinas et al., 2013). The authors further demonstrated that CBD treatment of GBM cells significantly downregulated major proteins associated with tumor invasion, in particular MMP-9 and TIMP-4 (Solinas et al., 2013). Moreover additional MMPs and TIMPs have been linked to the anti-invasive effects of cannabinoids in glioma. Specifically, both TIMP-1 and MMP-2 were downregulated by THC treatment of glioma cells. These effects were mediated via ceramide accumulation and activation of p8 stress protein and, interestingly, were observed in glioma bearing mice as well as in two patients with recurrent GBM who had received intra-tumor injections with THC (Blázquez et al., 2008a,b).

## Cannabinoids and Glioma Stem-Like Cells (GSCs)

A major challenge for GBM treatment is the resistance of the recurrent tumor to therapy. Accumulating evidence indicates that a subpopulation of GSCs contributes to this phenomenon through multiple mechanisms, such as alteration of DNA damage response, hypoxic microenvironment, Notch signaling pathway or multidrug resistance (reviewed in Liebelt et al., 2016).

GSCs express both major cannabinoid receptors, CB1 and CB2, as well as other components of the endocannabinoid system (Aguado et al., 2007). Exploratory gene array studies found that cannabinoid agonists altered the expression of genes involved in stem cell proliferation and differentiation. Cannabinoid-treated GSCs responded with increased S-100ß and GFAP expression and with simultaneous downregulation of the neuroepithelial progenitor marker nestin. Furthermore, cannabinoid challenge reduced the efficiency of GSCs to initiate glioma formation *in vivo*, as indicated by decreased neurosphere formation and cell proliferation in secondary xenografts (Aguado et al., 2007). The differentiation of GSCs was recently linked to the expression levels of the transcription factor Aml-1a. Nabissi et al. (2015) found that Aml-1a was upregulated during GSCs differentiation while Aml-1a knock-down restored a stem-cell phenotype in differentiated GSCs. Interestingly, treatment of GSCs with CBD upregulated the expression of Aml-1a in a TRPV2- and PI3K/Akt-dependent manner thereby inducing autophagy and abrogating the chemoresistance of GSCs to BCNU therapy (Nabissi et al., 2015).

Another potential mechanism regulating the “stemness” of GSCs upon cannabinoid treatment involves the intracellular increase of ROS. Specifically, CBD was shown to inhibit the self-renewal of GSCs via activation of the p38-MAPK pathway and downregulation of key stem cell mediators such as Sox2, Id1 and p-STAT3, while co-treatment with antioxidants abrogated these effects. *In vivo*, treatment of intracranial GSCs-derived tumors with CBD inhibited tumor cell proliferation, activated the pro-apoptotic caspase-3 and significantly prolonged the survival of tumor-bearing mice. Even though a subset of GSCs adapted to CBD treatment and led to tumor regrowth, this phenomenon could be abrogated by combined therapy with CBD and small molecule modulators of ROS (Singer et al., 2015).

## Clinical Relevance and Future Perspective of Cannabinoids in GBM Therapy

The antineoplastic effects of cannabinoids have been investigated in a number of *in vitro* and *in vivo* studies (reviewed in Ladin et al., 2016). A pilot phase I clinical trial for the treatment of GBM patients indicated a good safety profile for THC (Velasco et al., 2007). The intra-tumor administration of THC in nine patients with actively growing recurrent GBM decreased tumor cell proliferation (Guzmán et al., 2006) and induced apoptosis (Carracedo et al., 2006). In contrast, cannabinoids promoted the survival of healthy oligodendrocytes (Molina-Holgado et al., 2002), astrocytes (Gómez Del Pulgar et al., 2002), and neurons (Howlett et al., 2002; Mechoulam, 2002). A tumor-specific cytostatic/cytotoxic effect of cannabinoids would, therefore, have great relevance for the treatment of GBM.

Pre-clinical studies have also investigated the anti-tumor effects of cannabinoid combinations (in particular THC:CBD) and found that the anti-neoplastic effect of THC was enhanced when combined with CBD (reviewed in Ladin et al., 2016). The therapeutic potential of THC:CBD combinations was, furthermore, tested in combination with standard GBM chemotherapy, such as the alkylating anti-neoplastic drug TMZ or with ionizing radiotherapy. In a GBM xenograft model in nude mice, the reduction of tumor size could be enhanced by co-administration of THC with CBD and TMZ in comparison to the effects of THC, CBD and TMZ alone (Torres et al., 2011). In a further study, THC:CBD co-treatment of orthotopic GBM tumors in C57BL/6 mice enhanced the killing effect of ionizing radiation (Scott et al., 2014; Ladin et al., 2016).

These beneficial effects of THC:CBD preparations in pre-clinical models have led to a placebo-controlled phase II clinical trial investigating a THC:CBD mixture in combination with dose-intense TMZ in GBM patients (clinical trial NCT01812603). The company GW Pharmaceuticals reported in their orphan drug-designated study positive results in the treatment of GBM (Schultz and Beyer, 2017; Schultz, 2018). This study included 21 adult patients with histopathologically-confirmed GBM and with a Karnofsky performance scale of 60% or greater (clinical trial NCT01812603; Schultz and Beyer, 2017). Patients received orally a maximum of 12 sprays per day delivering 100 μl of a solution containing 27 mg/ml THC and 25 mg/ml CBD. The control group received TMZ only and had a 44% 1-year survival rate. In contrast the THC:CBD plus TMZ group showed a 83% 1-year survival rate with a median survival over 662 days compared with 369 days in the control group. (Schultz and Beyer, 2017; Schultz, 2018). These first results of clinical investigations are promising and point to the importance of cannabinoid translational research leading to clinically relevant studies. In the future, endocannabinoid-degrading MAGL enzyme might also be an interesting target since it changes the fatty acid network of cancer cells modulating their pathogenicity (Nomura et al., 2010).

In conclusion, cannabinoids show promising anti-neoplastic functions in GBM by targeting multiple cancer hallmarks such as resistance to programmed cell death, neoangiogenesis, tissue invasion or stem cell-induced replicative immortality. The effects of cannabinoids can be potentially enhanced by combination of different cannabinoids with each other or with chemotherapeutic agents. This requires, however, a detailed understanding of cannabinoid-induced molecular mechanisms and pharmacological effects. Ultimately, these findings might foster the development of improved therapeutic strategies against GBM and, perhaps, other diseases of the nervous system as well.

## Author Contributions

CD, IES and MK wrote the manuscript.

## Conflict of Interest Statement

The authors declare that the research was conducted in the absence of any commercial or financial relationships that could be construed as a potential conflict of interest.
